# TIAM1 acts as an actin organization regulator to control adipose tissue–derived pericyte cell fate

**DOI:** 10.1172/jci.insight.159141

**Published:** 2023-07-10

**Authors:** Ginny Ching-Yun Hsu, Yiyun Wang, Amy Z. Lu, Mario A. Gomez-Salazar, Jiajia Xu, Dongqing Li, Carolyn Meyers, Stefano Negri, Sintawat Wangsiricharoen, Kristen Broderick, Bruno Peault, Carol Morris, Aaron W. James

**Affiliations:** 1 Department of Pathology, Johns Hopkins University, Baltimore, Maryland, USA.; 2 Department of Orthodontics, Oregon Health & Science University, Portland, Oregon, USA.; 3 Department of Plastic Surgery, Johns Hopkins University, Baltimore, Maryland, USA.; 4 UCLA and Orthopaedic Hospital Department of Orthopaedic Surgery and Orthopaedic Hospital Research Center, Los Angeles, California, USA.; 5 Center for Cardiovascular Sciences, University of Edinburgh, Edinburgh, United Kingdom.; 6 Department of Orthopaedic Surgery, Johns Hopkins University, Baltimore, Maryland, USA.

**Keywords:** Bone Biology, Cell Biology, Adipose tissue, Bone disease, Pericytes

## Abstract

Pericytes are multipotent mesenchymal precursor cells that demonstrate tissue-specific properties. In this study, by comparing human adipose tissue– and periosteum-derived pericyte microarrays, we identified T cell lymphoma invasion and metastasis 1 (TIAM1) as a key regulator of cell morphology and differentiation decisions. TIAM1 represented a tissue-specific determinant between predispositions for adipocytic versus osteoblastic differentiation in human adipose tissue–derived pericytes. *TIAM1* overexpression promoted an adipogenic phenotype, whereas its downregulation amplified osteogenic differentiation. These results were replicated in vivo, in which *TIAM1* misexpression altered bone or adipose tissue generation in an intramuscular xenograft animal model. Changes in pericyte differentiation potential induced by *TIAM1* misexpression correlated with actin organization and altered cytoskeletal morphology. Small molecule inhibitors of either small GTPase Rac1 or RhoA/ROCK signaling reversed TIAM1-induced morphology and differentiation in pericytes. In summary, our results demonstrate that TIAM1 regulates the cellular morphology and differentiation potential of human pericytes, representing a molecular switch between osteogenic and adipogenic cell fates.

## Graphical abstract

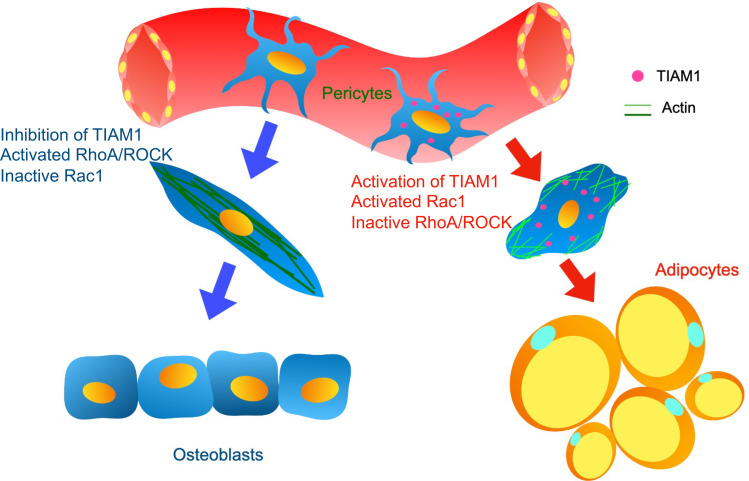

## Introduction

Pericytes have mesenchymal stem cell–like (MSC-like) properties, including multipotentiality, immunoregulatory functions, and diverse roles in tissue repair (1–3). A general hypothesis holds that pericytes function as tissue-specific progenitor cells during tissue regeneration (4). For example, skeletal pericytes can differentiate into osteoblasts, while smooth muscle–derived pericytes do not (5). Likewise, cord blood mesenchymal progenitors are more multipotent than postnatal bone marrow MSCs to undergo osteogenic differentiation, chondrogenesis, and adipogenesis (6). Pericytes derived from tissue around the joint demonstrate heightened chondrogenic potential (7). Important examples of heterologous differentiation of pericytes are also reported, and our group has focused on “adipose-to-bone” transdifferentiation of human pericytes (reviewed in ref. 8). Further exploration of human pericytes as tissue-specific progenitors and the cell- and context-specific permissive situations for heterologous differentiation have important implications for cell biology and the use of pericytes in cell therapy.

In previous work, we examined the tissue-specific properties of human pericytes by directly comparing pericytes from skeletal (periosteal) and adipose tissue sources (4). Results showed that CD146^+^Lin^–^ skeletal pericytes preferentially mineralized and ossified, whereas CD146^+^Lin^–^ adipose tissue pericytes preferentially formed adipocytes (4). Using transcriptomic analysis, we investigated osteogenesis-related signaling pathways and differentially expressed genes (DEGs) in pericytes from each tissue depot. Among other findings, we identified a subset of CXCR4-expressing pericytes in adipose tissue that displayed osteoprogenitor cell attributes (4). Due to these results, it has been suggested that specific genes and pathways maintain tissue-specific potential.

In the present study, we addressed the question of whether endogenous factors or signaling pathways within human adipose tissue pericytes restrain their ability to ossify. We reasoned that basal signaling pathways that actively inhibit osteogenic pathways may be present among adipose tissue pericytes in vivo. To this end, transcriptomic data of skeletal versus soft tissue CD146^+^ human pericytes were reanalyzed to identify differences among many pathways related to cytoskeletal organization and Rho GTPase signaling. From these DEGs, T cell lymphoma invasion and metastasis 1 (TIAM1) was chosen as a candidate. TIAM1 is a guanine nucleotide exchange factor (GEF) and can specifically regulate the Rho family of small GTPases (Rac, Rho, and Cdc42), including Rac1 (9). TIAM1 has been studied in contexts related to cell migration, adhesion, and proliferation by controlling actin remodeling (10, 11), which in turn is known to influence mesenchymal progenitor cell differentiation (12, 13).

Here, we employed a detailed examination of TIAM1 expression within human pericytes. Briefly, we observed that *TIAM1* misexpression alters pericyte cellular morphology leading to skewed osteo/adipogenic differentiation via changes in the reciprocal relationship of Rac1 and RhoA/ROCK signaling. This observation was recapitulated after xenotransplantation in mice. By using Rac1 or RhoA/ROCK signaling small molecular inhibitors NSC23766 or Y27632, the reverse changes in pericyte morphology and differentiation were observed. This finding demonstrates that TIAM1 directs cell differentiation, an important feature in human pericytes that may explain tissue-intrinsic differences in this progenitor cell type.

## Results

### TIAM1 is enriched within adipose tissue–derived pericytes.

**Figure 1:**
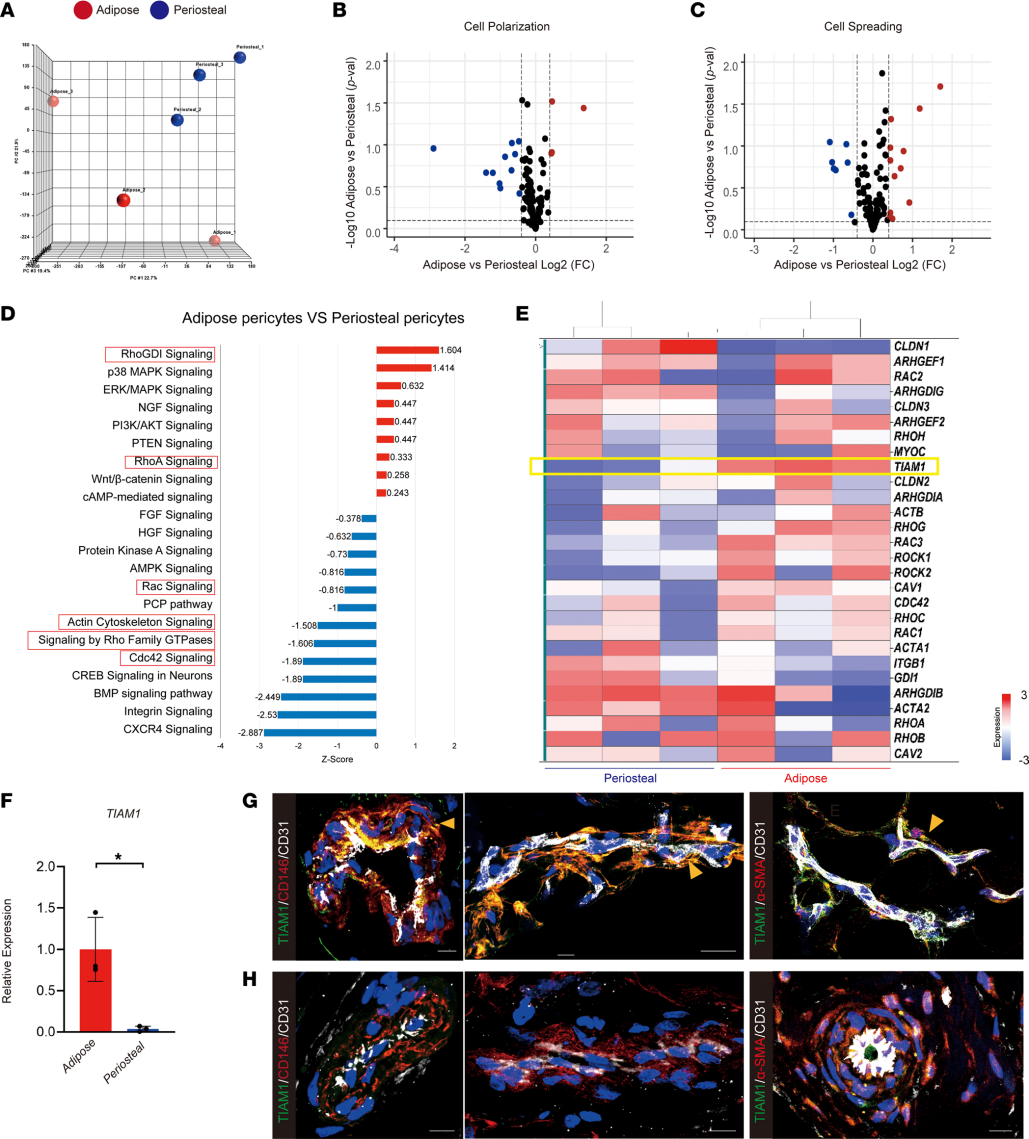
TIAM1 is highly expressed in human pericytes within adipose tissue but not skeletal tissue. (**A**–**E**) Transcriptome of skeletal (periosteal) and adipose CD146^+^ pericytes, by Affymetrix Clariom D microarray among undifferentiated, culture-expanded cells of equal passage. (**A**) Principal component analysis among periosteal and adipose CD146^+^ pericytes. Each dot represents pericytes from different patients. (**B** and **C**) Volcano plots involving genes associated with cell polarization (**B**) and cell spreading (**C**) among adipose and periosteal pericytes. Red dots indicate transcripts enriched in adipose pericytes, whereas blue dots indicate transcripts enriched in periosteal pericytes. (**D**) Ingenuity Pathway Analysis (IPA) demonstrating differentially activated pathways among adipose versus periosteal CD146^+^ pericytes. Red boxes indicate pathways of interest in cytoskeletal organization. (**E**) Heatmap of genes associated with Rho GTPases, including *TIAM1*. (**F**) Validation of differential *TIAM1* expression among CD146^+^ pericytes from adipose and periosteal sources, by qRT-PCR. (**G**) Immunofluorescence staining of TIAM1 in microvessels of subcutaneous human adipose tissue. (**H**) Immunofluorescence staining of TIAM1 in microvessels of human periosteum. Co-immunostaining for pericyte markers (CD146 and α-SMA) and CD31 was performed in **G** and **H**. Yellow arrow indicates colocalization of CD146 or α-SMA (red) and TIAM1 (green) immunoreactivity. Endothelium (CD31) appears white. Nuclear counterstain appears blue. Scale bars: 10 μm. *P < 0.05. Statistical analysis was performed using a 2-sample Student’s *t* test.

To investigate tissue-specific gene expression of pericytes, human adipose and periosteal pericytes were FACS-sorted to obtain CD146^+^CD31^–^CD45^–^ cell preparations (Supplemental Figure 1; supplemental material available online with this article; https://doi.org/10.1172/jci.insight.159141DS1) (4). A clear separation between gene expression profiles was observed when comparing periosteal and adipose pericytes, as revealed by principal component analysis (Figure 1A). Gene Ontology (GO) reanalysis revealed changes in terms related to cell size, shape, and spreading between adipose and periosteal pericytes. For example, enrichment in genes associated with cell polarization was identified among periosteal pericytes (Figure 1B and Supplemental Table 3). On the other hand, GO terms such as cell spreading were enriched in adipose tissue–derived pericytes (Figure 1C and Supplemental Table 4). Ingenuity Pathway Analysis (IPA; QIAGEN) showed a series of Rho GTPase–related cytoskeletal pathways that were differentially activated within adipose versus periosteal CD146^+^ pericytes, such as the RhGDI pathway, RhoA and Rac signaling pathways, as well as terms such as actin cytoskeletal signaling (Figure 1D, red boxes). Among single genes involved in Rho GTPase–related signaling, the Rho regulator TIAM1 showed enrichment in expression across adipose tissue–derived, but not skeletally derived pericytes (Figure 1E).

TIAM1 is a main regulator of the Rho proteins connecting extracellular signals to cytoskeletal modifications (14–18). For instance, TIAM1 regulates integrin-mediated cell-matrix adhesion, E-cadherin cell adhesion, and cell polarity (11, 19). Differential gene expression of *TIAM1* in separately derived human pericyte samples was verified by real-time quantitative reverse transcription PCR (qRT-PCR) (Figure 1F). Results from immunohistochemical staining of TIAM1, pericyte markers CD146 and α–smooth muscle actin (α-SMA), and endothelial marker CD31 showed that TIAM1 was enriched in adipose tissue pericytes, which was verified by a high degree of overlap between TIAM1 and known pericyte markers (CD146 and α-SMA) (Figure 1, G and H). Results showed that TIAM1 was enriched in adipose tissue pericytes, which was verified by a high degree of overlap between TIAM1 and known pericyte markers (CD146 and α-SMA) (Figure 1G). In contrast, immunohistochemical staining of blood vessels in human periosteal tissues revealed a relatively limited TIAM1 immunoreactivity among bone-associated pericytes (Figure 1H).

### TIAM1 regulates adipogenic versus osteogenic cell fates among human pericytes.

**Figure 2:**
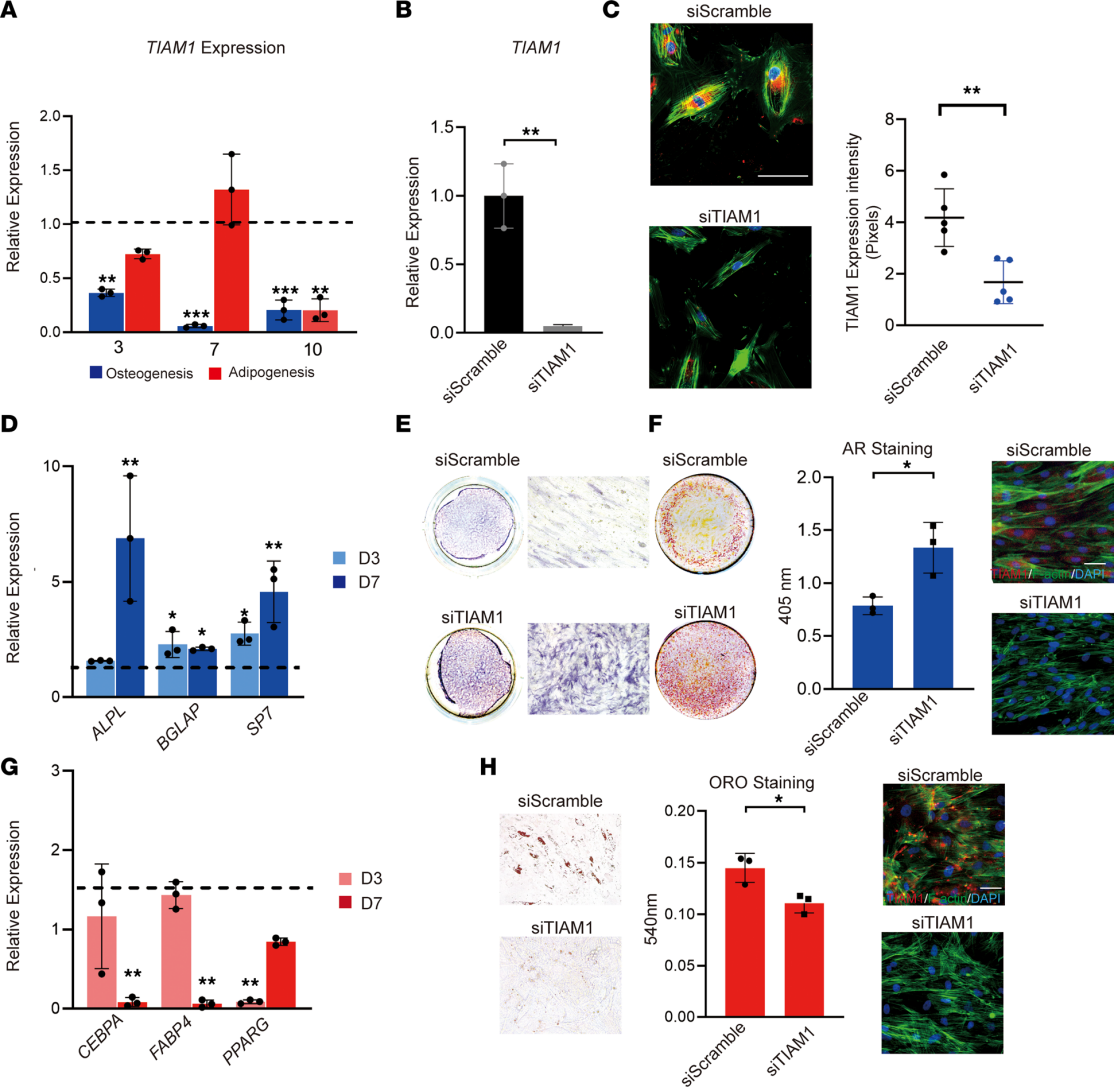
*TIAM1* knockdown favors osteogenic over adipogenic differentiation of human adipose CD146^+^ pericytes. Human pericytes from subcutaneous white adipose tissue were FACS-isolated and cultured under osteogenic to adipogenic differentiation conditions. (**A**) *TIAM1* expression during osteogenic and adipogenic differentiation of human pericytes, as assessed by qRT-PCR. Dashed lines indicate expression among undifferentiated cells. (**B**) Knockdown (KD) efficiency by qRT-PCR 48 hours after treatment with *TIAM1* siRNA or scramble siRNA. (**C**) KD efficiency by fluorescence immunocytochemistry for TIAM1 and semiquantitative analysis 48 hours after KD. TIAM1 immunostaining appears red, while F-actin staining appears green. Random images (*N* = 5) of fluorescence staining were obtained for semiquantitation of TIAM1 (red) or F-actin (green) area (mean red or green fluorescent area per field view). (**D**) Osteogenic gene markers at days 3 and 7 of osteogenic differentiation among *TIAM1*-KD pericytes, assessed by qRT-PCR. Dashed lines indicate expression among scramble siRNA at the same time point. ALPL, alkaline phosphatase; BGLAP, osteocalcin; SP7, osterix. (**E**) Alkaline phosphatase (ALP) staining shown by whole-well and representative 10× original magnification microscopic images, day 7 of differentiation. (**F**) Alizarin red staining with quantification on day 10 of osteogenic differentiation, shown by whole-well images. Immunohistochemistry showed the TIAM1 and F-actin expression during osteogenesis. (**G**) Adipogenic gene marker expression at days 3 and 7 of adipogenic differentiation among *TIAM1*-KD pericytes, assessed by qRT-PCR. Dashed lines indicate expression among scramble siRNA at the same time point. CEBPA, CCAAT enhancer binding protein alpha; FABP4, fatty acid-binding protein 4; PPARG, peroxisome proliferator–activated receptor gamma. (**H**) Oil Red O staining and quantification, day 10 of adipogenic differentiation (representative 10× original magnification images shown). Immunohistochemistry showed the TIAM1 and F-actin expression during adipogenesis. *P < 0.05; **P < 0.01 in comparison with scramble siRNA at the corresponding time point. Experiments performed in at least triplicate experimental replicates. Statistical analysis was performed using a 2-sample Student’s *t* test or 1-way ANOVA test (3+ groups), followed by Tukey’s post hoc test. White scale bars: 20 μm.

Data demonstrated that *TIAM1* expression decreased across time points in osteogenic medium (Figure 2A, blue bars). In contrast, no significant difference in *TIAM1* expression was observed until late adipogenesis, at which time *TIAM1* expression levels were reduced in comparison with undifferentiated cells (Figure 2A, red bars). Next, the effects of *TIAM1* knockdown (KD) on pericyte differentiation were assessed. Validation of siRNA-mediated KD was first performed by qRT-PCR (Figure 2B) and immunocytochemistry and semiquantitative analysis (Figure 2C). Under osteogenic differentiation conditions, *TIAM1* KD led to an increase in osteogenic gene expression at both 3 and 7 days of differentiation, including alkaline phosphatase (ALP or ALPL), osteocalcin (BGLAP), and osterix (SP7) (Figure 2D). Consistent with gene expression studies, staining of ALP enzymatic activity was significantly increased with *TIAM1* KD at 10 days of differentiation (Figure 2E). Moreover, bone nodule deposition assessed by alizarin red (AR) staining and photometric quantification showed a robust increase in the mineralization of cells with *TIAM1* KD (Figure 2F). In contrast, *TIAM1* KD demonstrated opposing effects on adipogenic differentiation of human pericytes (Figure 2, G and H). This included significantly decreased expression of adipogenic differentiation markers by qRT-PCR, including peroxisome proliferator–activated receptor gamma (*PPARG*) at 3 days, as well as CCAAT enhancer binding protein alpha (*CEBPA*) and fatty acid–binding protein 4 (*FABP4*) at 7 days of adipogenic differentiation (Figure 2G). Likewise, *TIAM1* KD led to reduced intracellular lipid droplets as visualized by Oil Red O staining (Figure 2H). Changes in differentiation potential with *TIAM1* KD were not accompanied by changes in proliferation, as observed by MTS assays (Supplemental Figure 2A) (Promega). The same trends in favoring osteogenic differentiation over adipogeneic differentiation were observed in *TIAM1*-KD periosteal pericytes (Supplemental Figure 3).

**Figure 3:**
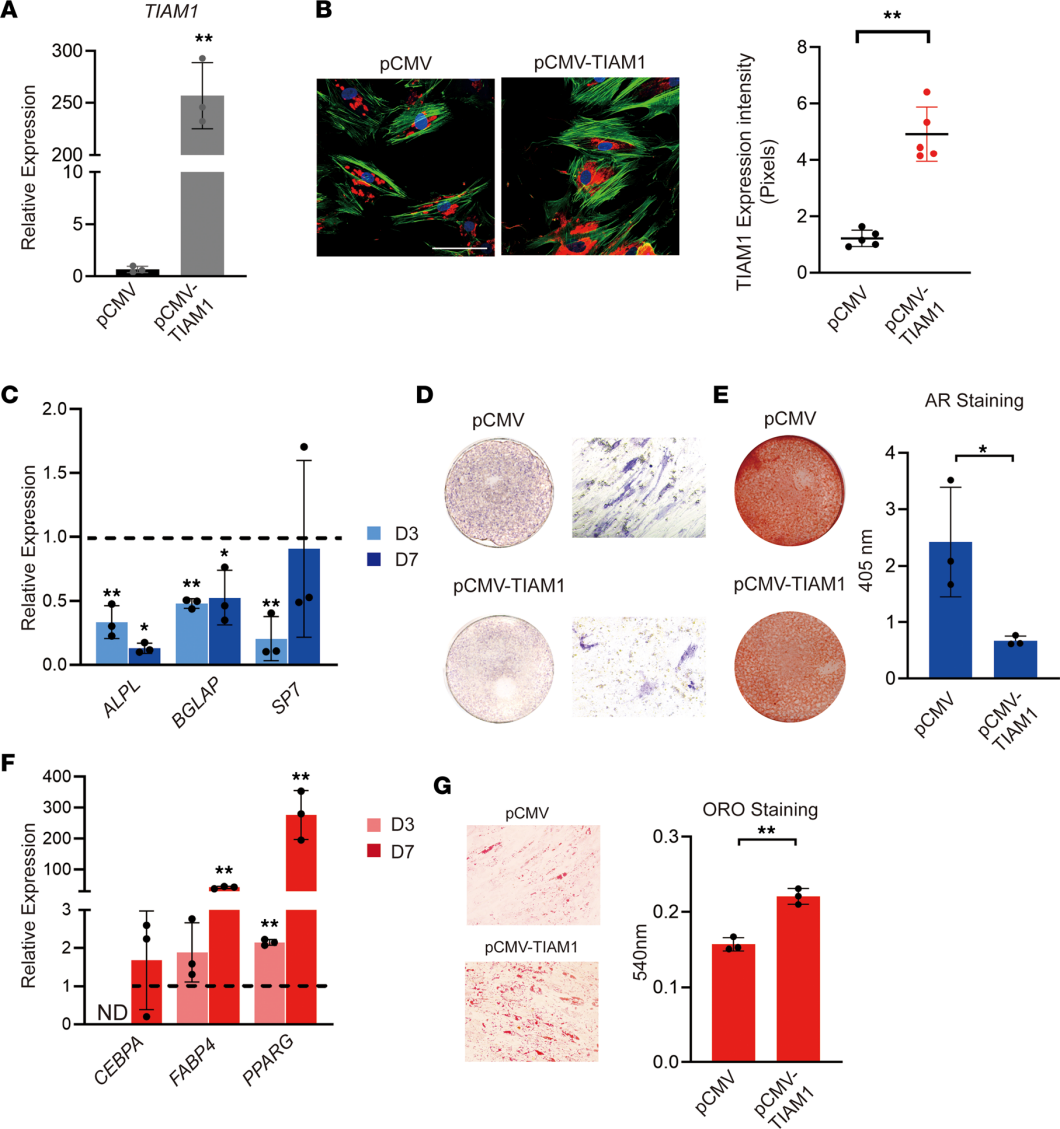
*TIAM1* overexpression favors adipogenic over osteogenic differentiation of human adipose tissue–derived CD146^+^ pericytes. (**A**) Validation of *TIAM1* overexpression (OE) by qRT-PCR, performed 48 hours after treatment with *TIAM1* ORF plasmid (pCMV-TIAM1) or vector control (pCMV). (**B**) Validation of *TIAM1* OE by fluorescence immunocytochemistry for TIAM1 and semiquantitative analysis, after 48 hours. TIAM1 immunostaining appears red, while F-actin staining appears green. Random images (*N* = 5) of fluorescence staining were obtained for semiquantitation of TIAM1 (red) or F-actin (green) area (mean red or green fluorescence area per field view). (**C**) Osteogenic gene markers at days 3 and 7 of osteogenic differentiation among *TIAM1* OE pericytes, assessed by qRT-PCR. Dashed lines indicate expression among pCMV vector control at the same time point. ALPL, alkaline phosphatase; BGLAP, osteocalcin; SP7, osterix. (**D**) Alkaline phosphatase (ALP) staining shown by whole-well and representative 10× original magnification microscopic images on day 7 of differentiation. (**E**) Alizarin red staining with quantification on day 10 of osteogenic differentiation, shown by whole-well images. (**F**) Adipogenic gene marker expression at days 3 and 7 of adipogenic differentiation among *TIAM1* OE pericytes, assessed by qRT-PCR. Dashed lines indicate expression among vector control at the same time point. CEBPA, CCAAT enhancer binding protein alpha; FABP4, fatty acid–binding protein 4; ND, not detected; PPARG, peroxisome proliferator–activated receptor gamma. (**G**) Oil Red O staining and quantification on day 10 of adipogenic differentiation (representative 10× original magnification images shown). *P < 0.05; **P < 0.01 in comparison with pCMV vector control at the corresponding time point. Experiments performed in at least triplicate experimental replicates. Statistical analysis was performed using a 2-sample Student’s *t* test. White scale bars: 20 μm.

Transfection of a CMV plasmid (pCMV) control used to promote *TIAM1* overexpression led to a 250-fold upregulation of *TIAM1* expression by qRT-PCR (Figure 3A), which was validated by immunocytochemical staining and quantification (Figure 3B). *TIAM1* overexpression in pericytes under osteogenic differentiation conditions led to a decrease in the expression of osteogenic gene markers in comparison with the control plasmid (Figure 3C). Inhibition of osteogenic differentiation with *TIAM1* overexpression was validated using ALP and AR staining (Figure 3, D and E). *TIAM1* overexpression led to a significant increase in the expression of adipogenic gene markers including *CEBPA*, *FABP4*, and *PPARG* (Figure 3F). With *TIAM1* overexpression, Oil Red O staining likewise showed a significant increase in the intracellular accumulation of lipids after 10 days of differentiation (Figure 3G). Changes in differentiation potential with *TIAM1* overexpression were not accompanied by changes in proliferation (Supplemental Figure 2B). In summary, our studies thus far suggest that TIAM1 exerts prominent pro-adipogenic/anti-osteogenic effects in human pericytes without affecting cell proliferation.

### TIAM1 misexpression alters human pericyte morphology.

**Figure 4:**
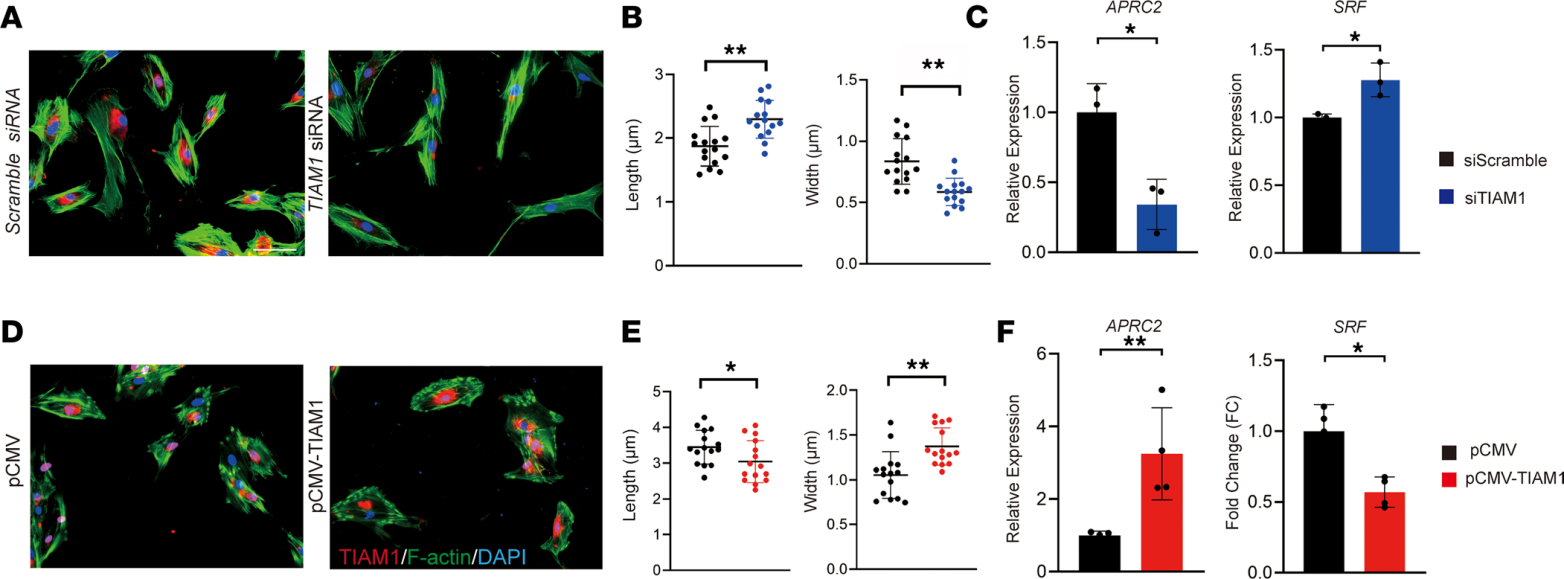
*TIAM1* misexpression alters pericyte cellular morphology and gene targets of Rac1 and RhoA/ROCK signaling. (**A**) Cellular morphology of human pericytes 48 hours after treatment with *TIAM1* siRNA or scramble siRNA. F-actin staining appears green, while TIAM1 immunostaining appears red. (**B**) Quantification of F-actin cell length and cell width among *TIAM1* siRNA– or scramble siRNA–treated human pericytes. Each dot represents an individual cell, with images obtained from 3 random high-magnification fields from each group. (**C**) Specific target gene expression for Rac1 (*ARPC2*) and RhoA/ROCK (*SRF*) signaling pathways among *TIAM1* siRNA– or scramble siRNA–treated human pericytes, 24 hours after treatment by qRT-PCR. *ARPC2*, actin related protein 2/3 complex subunit 2; *SRF*, serum response factor. (**D**) Cellular morphology of human pericytes 48 hours after incubation with *TIAM1* or control plasmids. F-actin staining appears green, while TIAM1 immunostaining appears red. (**E**) Quantification of F-actin angulation, cell length, and cell width among human pericytes treated with *TIAM1* or control plasmid. Each dot represents an individual cell, with images obtained from 3 random high-magnification fields from each group. (**F**) Specific target gene expression for Rac1 (*ARPC2*) and RhoA/ROCK (*SRF*) pathways, by qRT-PCR, 48 hours after treatment. *P < 0.05; **P < 0.01. Statistical analysis was performed using a 2-sample Student’s *t* test. White scale bars: 20 μm.

Silencing of *TIAM1* gene expression led to an elongated and spindled cell morphology in comparison with an siRNA control, as visualized using F-actin staining (Figure 4A). When quantified, *TIAM1*-KD pericytes showed increased length and decreased width (Figure 4B). Target genes for Rac1 and RhoA/ROCK signaling were next assessed, including actin related protein 2/3 complex subunit 2 (*ARPC2*) and serum response factor (*SRF*), respectively (Figure 4C). As expected from its known function in other cell types (20), *TIAM1* when silenced led to a significant increase in RhoA/ROCK and reduction in Rac1 gene targets (Figure 4C). Converse experiments were performed in the context of *TIAM1* overexpression (Figure 4, D–F). TIAM1 overexpression led to a wider cell shape in human pericytes (Figure 4D), which when quantified by cell, showed an overall reduced cell length and increased cell width (Figure 4E). In parallel, *TIAM1* overexpression led to an increase in the Rac1 gene marker *ARPC2* and a significant decrease in *SRF* expression (Figure 4F). Thus, *TIAM1* gene misexpression alters actin organization and cellular shape in human pericytes and is associated with changes in Rac1/RhoA/ROCK signaling activity.

### TIAM1 regulates pericyte cytoskeletal remodeling and cellular differentiation via Rac1 and RhoA/ROCK signaling.

**Figure 5:**
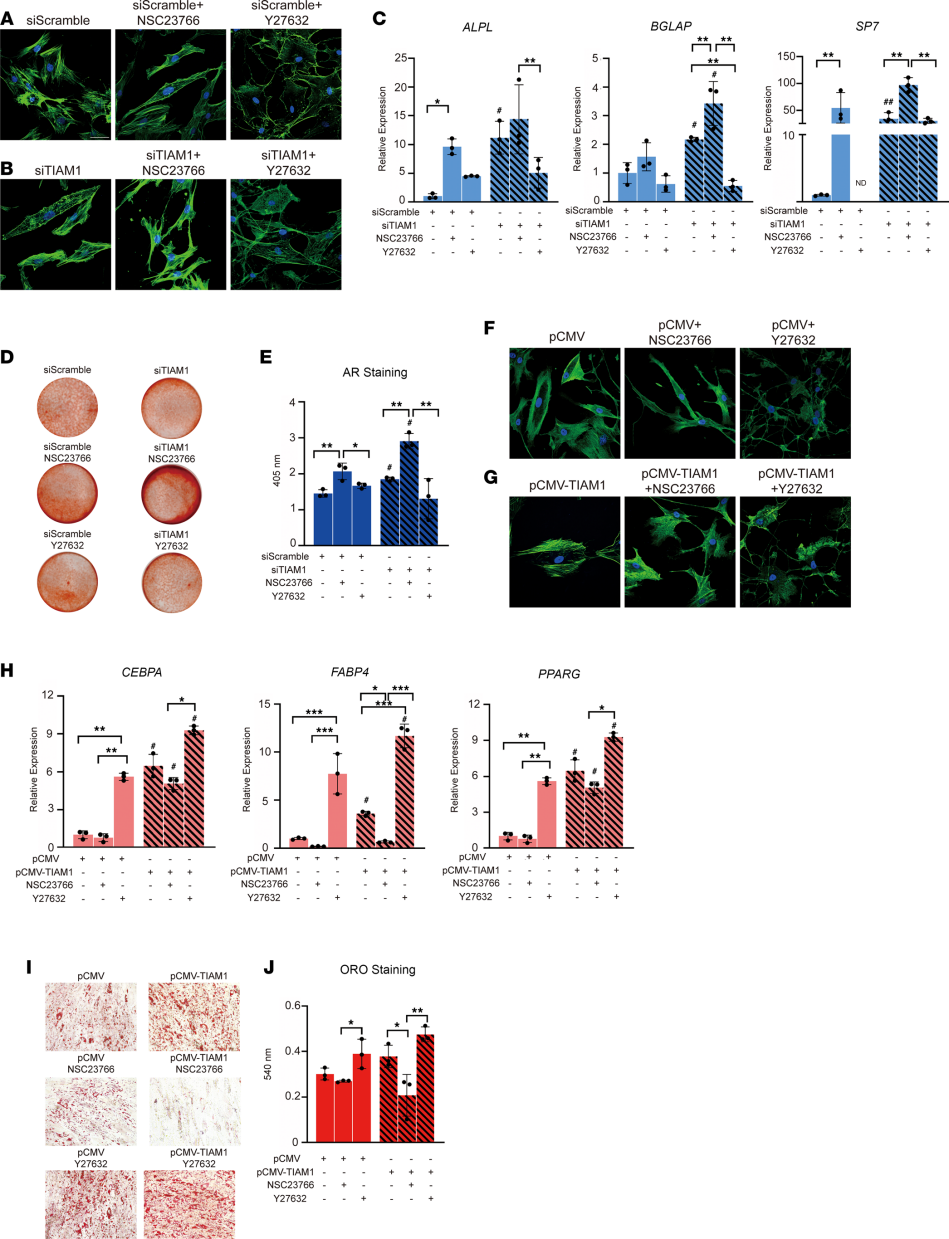
Rac1 and ROCK inhibitors alter pericyte morphology and osteo/adipogenic differentiation potential with *TIAM1* misexpression. (**A** and **B**) Pericyte morphology after 48 hours of treatment with NSC23766 (Rac1 inhibitor, 5 μM) or Y27632 (ROCK inhibitor, 10 μM). F-actin appears green and DAPI staining appears blue. Images shown of human pericytes with *TIAM1*-KD or scramble siRNA. (**C**) Osteogenic gene markers at day 7 of osteogenic differentiation among *TIAM1*-KD or siRNA control–treated pericytes with NSC23766 or Y27632, as assessed by qRT-PCR. ALPL, alkaline phosphatase; BGLAP, osteocalcin; SP7, osterix. (**D** and **E**) Alizarin red staining and quantification on day 10 of osteogenic differentiation. (**F** and **G**) Pericyte morphology with treatment of NSC23766 (5 μM) or Y27632 (10 μM). F-actin appears green and DAPI staining appears blue. Images shown of human pericytes with *TIAM1* OE or vector control. (**H**) Adipogenic gene marker expression at day 7 of adipogenic differentiation among *TIAM1* OE or vector control–treated pericytes with NSC23766 or Y27632, as assessed by qRT-PCR. CEBPA, CCAAT enhancer binding protein alpha; FABP4, fatty acid-binding protein 4; ND, not determined; PPARG, peroxisome proliferator–activated receptor gamma. (**I** and **J**) Oil Red O staining and quantification on day 10. Representative images shown at 10× original magnification. *P < 0.05; **P < 0.01; ***P < 0.005 between the groups. ^#^*P* < 0.05 in comparison with the corresponding treatment group with siScramble/pCMV control. Each dot in the scatterplots represents an individual well. Statistical analysis was performed using a 2-way ANOVA followed by Tukey’s post hoc test. White scale bars (**A**, **B**, **F**, and **G**): 20 μm.

Next, the Rac1 inhibitor NSC23766 or RhoA/ROCK inhibitor Y27632 was used as treatment to observe pericyte morphology and differentiation potential in the context of *TIAM1* gene misexpression (Figure 5). Cell morphology was first observed in growth medium (Figure 5, A and B). Under siRNA control conditions, pericytes treated with NSC23766 demonstrated a spindle cell shape, while pericytes treated with Y27632 demonstrated a round cell shape (Figure 5A). Under *TIAM1*-KD conditions, cell spindling was amplified under NSC23766-treated conditions, while Y27632 treatment reversed the spindled appearance induced by *TIAM1* silencing (Figure 5B). Recapitulating our prior findings, KD of *TIAM1* significantly increased the expression of osteogenic gene markers in human pericytes (Figure 5C). The Rac1 inhibitor NSC23766 further amplified the osteogenic effect of *TIAM1* KD, while the RhoA/ROCK inhibitor Y27632 partially or completely reversed this induction of osteogenic gene expression. Mineralization detection by AR staining and photometric quantification further validated these significant changes in osteogenic differentiation (Figure 5, D and E).

Converse experiments were next performed in the context of *TIAM1* overexpression and adipogenic differentiation with and without small molecule inhibitors (Figure 5, F–J). Substantial changes in cell shape were observed with the addition of small molecule inhibitors Rac1 or RhoA/ROCK (Figure 5, F and G). By specific adipogenic gene expression (Figure 5H) and Oil Red O staining and quantification (Figure 5, I and J), the Rac1 inhibitor NSC23766 partially or completely reversed an increase in adipogenesis under *TIAM1* overexpression conditions. Conversely, the RhoA/ROCK inhibitor increased adipogenesis under control conditions and additively increased adipogenic differentiation among *TIAM1*-overexpressing pericytes. Thus, co-application of small molecules in the context of TIAM1 misexpression indicates Rac1 and RhoA/ROCK signaling in TIAM1-directed differentiation of human pericytes.

### TIAM1 misexpression in human pericytes alters tissue generation in vivo.

**Figure 6:**
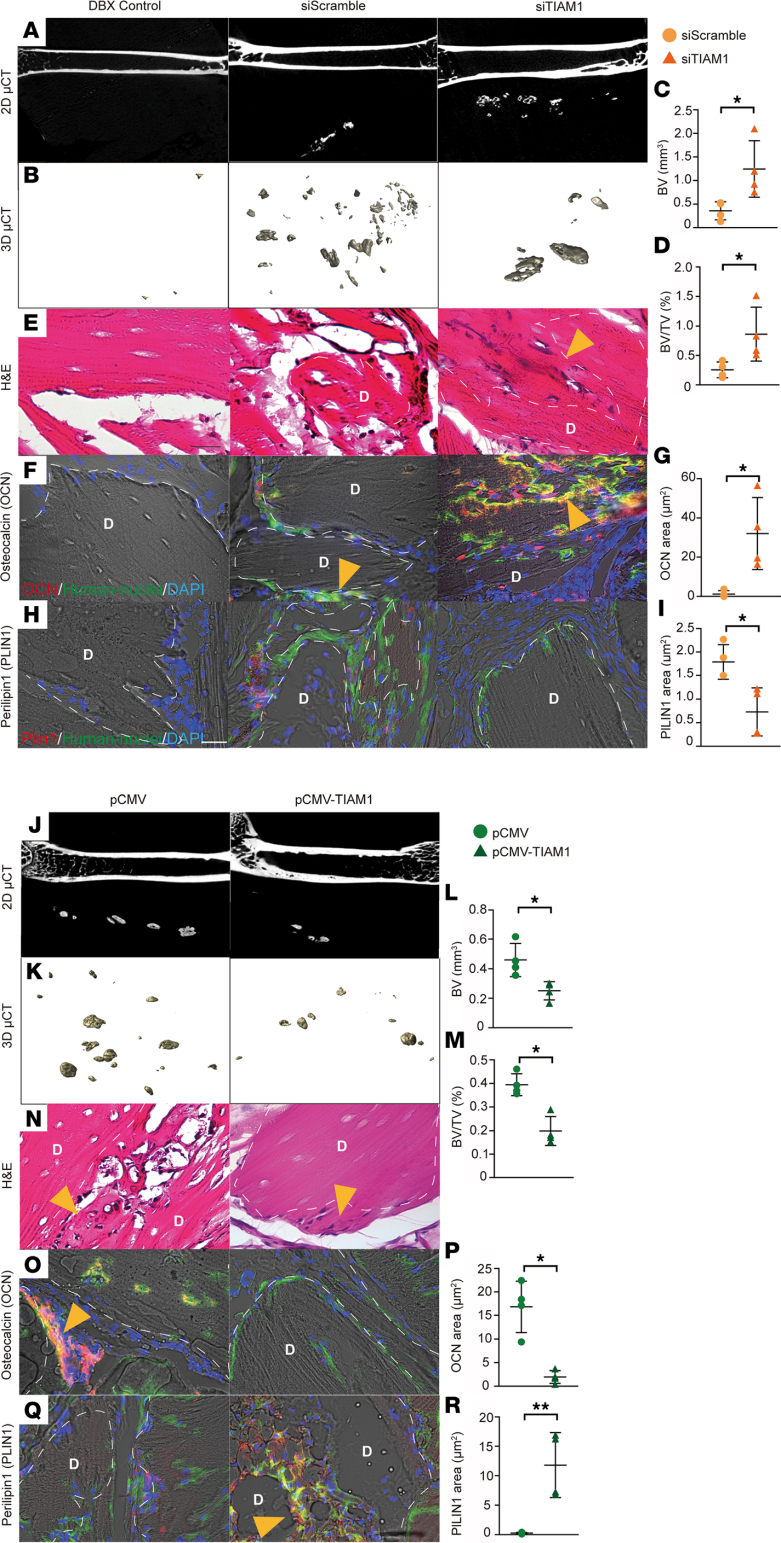
*TIAM1* misexpression alters bone and adipose tissue generation after human pericyte xenotransplantation. *TIAM1*-KD or -OE pericytes or indicated control were implanted subcutaneously in the dorsum of adult athymic nude male mice (Charles River Laboratories strain code: 490) using a demineralized bone matrix carrier (3 million cells/50 mg DBX). Assessments were performed after 8 weeks. (**A**–**K**) *TIAM1*-KD pericyte implants in relation to scramble siRNA pericytes. (**A**) Representative 2-dimensional μCT images, (**B**) 3-dimensional μCT reconstructions, and quantification of (**C**) bone volume (BV) and (**D**) fractional bone volume (BV/TV). Histology by (**E**) H&E staining. (**F**) Osteocalcin (OCN) immunohistochemistry in red and human nuclei (HuNu) in green, with (**G**) OCN quantification. (**H**) Perilipin 1 (Plin1) immunohistochemistry in red and HuNu in green (yellow arrow points out cell coexpression of both OCN and HuNu) and (**I**) Plin1 quantification. (**J**–**R**) *TIAM1* OE pericyte implants in relation to pCMV vector control pericytes. (**J**) Representative 2-dimensional μCT images, (**K**) 3-dimensional μCT reconstructions, and quantification of (**L**) BV and (**M**) BV/TV. Histology by (**N**) H&E staining. (**O**) OCN immunohistochemistry in red and HuNu in green, with (**P**) OCN quantification. (**Q**) Plin1 immunohistochemistry in red and HuNu in green, and (**R**) Plin1 quantification. Dotted white lines demarcate edges of the DBX area (“D”). Scale bar (all image panels): 20 μm. All quantitative data normalized to acellular control (DBX control). Each dot in the scatterplots represents an individual implantation site. *N* = 4 implants per group. *P < 0.05; **P < 0.01. Statistical analysis was performed using 2-sample Student’s *t* test.

Pericyte implantation studies were next performed in athymic mice over an 8-week period in the context of *TIAM1* misexpression. A significant increase in bone formation was observed among *TIAM1*-KD implantation sites, as observed by micro–computed tomography (μCT) cross-sectional imaging and reconstructions (Figure 6, A and B). Quantitative μCT analysis validated a significant increase in bone volume (BV) (Figure 6C) and fractional bone volume (BV/TV) (Figure 6D). Histology by H&E staining showed an increase in osteoblast activity within *TIAM1*-KD implants (Figure 6E). Likewise, immunohistochemistry for the terminally differentiated osteoblast marker osteocalcin (OCN) demonstrated increased antigen detection among *TIAM1*-KD implant sites (Figure 6, F and G). In these tissue sections, substantial coexpression with the human nuclear antigen was observed, demonstrating tissue generation by human cells (Figure 6, F and H). Immunohistochemistry for the adipocyte marker perilipin 1 (Plin1) demonstrated rare staining across tissue sections, which was primarily in control but not *TIAM1*-KD implantation sites (Figure 6, H and I).

Next, the converse effects of *TIAM1* overexpression in human pericytes on ectopic tissue generation were assessed in the same model (Figure 6, J–R). μCT imaging and analysis demonstrated a reduction in bone formation among pCMV-TIAM1 pericyte implant sites, including in cross-sectional imaging (Figure 6J), 3-dimensional μCT reconstructions (Figure 6K), and quantitative analysis (Figure 6, L and M). H&E staining validated the impression that new bone formation was reduced among *TIAM1* overexpression implant sites (Figure 6N). Foci of OCN immunofluorescence staining were present among control implantation sites and greatly reduced in *TIAM1* overexpression conditions (Figure 6, O and P). In contrast, the adipocyte marker Plin1 was significantly increased with adipocytes within pCMV-TIAM1–treated implant sites (Figure 6, Q and R) and showed notable overlap with human nuclear antigen expression. In summary, changes in *TIAM1* expression direct ectopic tissue generation between bone and fat tissue among human pericytes upon transplantation in a mouse model.

## Discussion

Mesenchymal progenitor cells are widely used for regenerative medicine for different pathologies, including bone conditions. Easily accessible tissues, such as adipose tissue, are one of the main sources of these cells. The inherent differentiation potential of progenitor cells is linked to their tissue of origin (4, 5). For instance, adipose tissue mesenchymal cells, such as pericytes, are prone to become adipocytes, whereas skeletally derived cells become bone (4). In the present study, the negative osteogenesis (pro-adipogenesis) regulator TIAM1 is identified, and its function is suggested to occur by regulating cytoskeletal morphology. Understanding tissue-specific regulators such as TIAM1 will help elucidate the mechanisms of progenitor cells in vivo and improve the use of these cells for therapeutic purposes. Through a series of transcriptomic, in vitro, and in vivo analyses, we identified TIAM1 expression in pericytes as a morphologic regulator that maintains tissue specificity within the adipose niche.

Pericytes located in the basement membrane are in contact with the endothelium of capillaries and, as such, are strongly influenced by a variety of physical stimuli such as fluid stress compression and microenvironmental tension (21). Studies have shown that changes in the differentiation potential of mesenchymal progenitor cells are dependent on tissue stiffness in vitro (22, 23). For example, progenitor cells cultured on gels demonstrate heightened chondrogenic and adipogenic differentiation potential compared with those cultured on stiff surfaces (22), and the use of supramolecular hydrogels at different stiffnesses determines neuronal, osteogenic, and chondrogenic differentiation (24). TIAM1 is a Rac1-specific GEF and negatively regulates RhoA/ROCK pathways. Rac1 and RhoA/ROCK are 2 reciprocal signaling pathways that regulate cell migration and proliferation through actin organization (25). Rac1 activation has been shown to induce membrane ruffles and lamellipodia in fibroblasts. In contrast, RhoA/ROCK has been suggested to increase differentiation in myogenesis and decrease adipogenesis via modulation of cytoskeletal tension and organization in pluripotent stem cells and fibroblasts (26). Small molecular inhibition of Rac1 and RhoA/ROCK pathway results in cytoskeletal arrangements that ultimately affect migration, proliferation, and adhesion (27–29). In the context of differentiation, prior reports have shown that high RhoA/ROCK activity is associated with osteogenic differentiation (26, 30), while a loss of RhoA and activation of Rac1 leads to an adipogenic phenotype (26, 31, 32). In this study, we verified these observations and further expanded the role that TIAM1 plays in modulating these signaling pathways to regulate cell morphology and, ultimately, cellular differentiation in pericytes. Cytoskeleton-dependent signaling pathways in pericytes regulate physical and chemical interconnections in the actin network, as well in the extracellular matrix (33). Actin assembly modifications play crucial roles in pericyte shape and contractility (33, 34). In the context of tissue specificity, the interplay of microenvironmental clues, such as extracellular matrix stiffness, may help determine cell phenotype and influence differentiation potential (35, 36). TIAM1 regulates the RhoA/ROCK family through the RhoA/ROCK inhibitor, Rac1, and serves as a regulator of tissue-specific pericytes between adipose tissue and bone. These reciprocal pathways may aid in changing MSC shape, which in turn can regulate the degree of osteogenic or adipogenic differentiation. The extent to which TIAM1 directly regulates adipogenesis and/or indirectly regulates adipogenesis through Rac1 or in combination with other Rac-associated pathways remains in question.

Important limitations exist in the study. Although TIAM1 is enriched in undifferentiated adipose-derived pericytes, as shown in our transcriptomic analyses, it is a curious finding that while TIAM1 remained unchanged during in vitro adipogenesis, its misexpression resulted in significant changes in the degree of adipogenic differentiation. Future studies could pursue in depth how TIAM1 directly or indirectly regulates adipogenesis. As a second limitation, the present study is shown only in adipose-derived CD146^+^ pericytes and has not been tested in other MSC types. In future studies, global or tissue-specific TIAM1-knockout mice may be better to elucidate the role of TIAM1 in pericyte differentiation decisions.

In conclusion, TIAM1 is a key regulator of cytoskeletal dynamics, affecting adipose-derived pericyte morphology and differentiation potential toward adipocytic versus osteoblastic fates. Studies on TIAM1 misexpression suggest a role for TIAM1 in tissue-specific pericyte differentiation decisions by regulating cytoskeletal morphology through Rac1 and RhoA/ROCK signaling pathways. This was demonstrated in xenograft animal models. Further study of TIAM1-Rac1 and RhoA/ROCK regulation of other tissue-specific mesenchymal precursor cell fates, and by extension development of new strategies for tissue engineering, will be worth future investigation.

## Methods

### Isolation and culture of periosteal or adipose CD146^+^ pericytes.

CD146^+^ pericytes were isolated from human adipose tissue and periosteum via FACS (4, 37, 38). *N* = 3 adipose and *N* = 3 periosteum samples were obtained from adult patient donors. Pericytes from 6 individual patients were collected for microarray, in vitro, and in vivo assays.

*N* = 3 adipose samples from 3 different patient sources were collected and analyzed by flow cytometry by the method described in a previous study (39, 40) (summary of antibodies presented in Supplemental Table 1). Briefly, passage 3 expanded CD146^+^ pericytes were analyzed by FlowJo software. In this manner, the FACS-purified CD146^+^ pericytes were snap-frozen for RNA isolation, culture-expanded to passages 3–8 for in vitro studies, or applied in a mouse intramuscular implantation model. For in vitro expansion, all cells were cultured in a nonclonal monolayer at 37°C in a humidified atmosphere containing 95% air and 5% CO_2_. FACS-purified CD146^+^ pericytes were cultured in DMEM with 10% fetal bovine serum (FBS) (Gibco) and 1% penicillin/streptomycin (Life Technologies). The medium was changed every 3 days, unless otherwise noted. Pericytes at passages 3 to 8 were used for in vitro and in vivo assays. Rac activity was inhibited using NSC23766 (Selleckchem), reconstituted in sterile water and used at a final concentration of 5 μM. ROCK signaling was inhibited using Y-27632 (Selleckchem), reconstituted in dimethyl sulfoxide (DMSO) and used at a final concentration of 10 μM. NSC23766 or Y27632 was added at cell seeding. For inhibitor studies, a DMSO vehicle control was present in all treatment groups.

### Microarray analysis.

The transcriptomes of *N* = 6 CD146^+^ periosteal and adipose pericytes from 6 different patients were examined by microarray (*N* = 3 periosteal and *N* = 3 adipose sources). Briefly, total RNA was extracted from passage 3 CD146^+^ pericytes by TRIzol (Life Technologies). After purification, the RNA samples were sent to the Johns Hopkins Medical Institute (JHMI) Transcriptomics and Deep Sequencing Core (Johns Hopkins University [JHU], Baltimore, Maryland, USA) for analysis using an Affymetrix Clariom D microarray. Microarray data were obtained from the NCBI’s Gene Expression Omnibus repository (accession number GSE125545). Data analyses were performed using software packages including Partek Genomics Suite, Spotfire DecisionSite with Functional Genomics, and QIAGEN IPA.

### Immunohistochemistry and microscopy.

For histology, 3 healthy human subcutaneous fat tissue samples were identified in our surgical pathology archives (JHU). Samples were obtained under IRB approval with a waiver of informed consent. Human fat tissues were embedded in optimal cutting temperature compound (Sakura) and cryosectioned at 20 μm thicknesses for immunofluorescence staining by methods described in the previous paper (antibodies used are listed in Supplemental Table 1) (4, 41). A ZEISS 800 confocal microscope was used for imaging immunofluorescence staining, or a Leica DM6 B microscope was used for imaging immunohistochemical staining.

### siRNA and transfection.

KD of *TIAM1* in CD146^+^ adipose pericytes was performed using Silencer Select chemically synthesized siRNA (Thermo Fisher Scientific, catalog 439824; S14138). Pericytes at passages 3–8 were seeded in 12-well plates at a density of 5 × 10^4^ cells per well. At 50% confluence, basal medium was replaced with antibiotic-free basal medium. Transfection was performed using X-tremeGENE siRNA Transfection Reagent (MilliporeSigma) and 150 pM *TIAM1-1* siRNA or scramble siRNA diluted in minimal essential medium (Opti-MEM) (42). To confirm siRNA efficiency, at 2 hours posttransfection, the medium was replaced with basal medium, and the efficiency of the KD was validated using qRT-PCR and immunocytochemistry (ICC).

### Plasmid-transfected overexpression.

*TIAM1* overexpression was assayed using a human *TIAM1* ORF mammalian expression plasmid (RG220233, OriGene). At 24 hours prior to transfection, CD146^+^ adipose pericytes at passages 3–8 were seeded in 12-well plates at a density of 5 × 10^4^ cells per well. Transfections were performed at 60% confluence; 1 μg of TIAM1 plasmid or control plasmid was mixed with 3 μL of Roche Xtreme gene HP transfection reagent in 100 μL of Opti-MEM and incubated at room temperature (RT) for 30 minutes. The DNA/transfection reagent mixture was then added in drops to wells (42). qRT-PCR and ICC were used to measure *TIAM1* gene expression and to confirm the efficacy of the plasmid.

### Osteogenic differentiation assays.

CD146^+^ adipose pericytes from 3 to 8 passages were seeded in 12-well plates at a density of 5 × 10^5^ cells per well. Osteogenic differentiation medium (ODM) consisted of DMEM, 10% FBS, 1% penicillin/streptomycin with 10 mmol/L β-glycerophosphate, 50 μmol/L ascorbic acid, and 1 mmol/L dexamethasone. At 24 hours after cell seeding, basal medium was replaced with ODM and replenished every 3 days.

For ALP and AR staining, cells were washed with PBS and fixed with 4% formaldehyde from 7 to 10 days of differentiation. Next, cells were stained with diazonium salt with 4% napthol AS-MX phosphate alkaline solution at RT for 15 minutes for ALP detection and with 2% AR solution at RT for 10 minutes for bone nodule deposition detection (4, 40, 42–44). Pictures were taken using an Olympus Epson scanner. For quantification, bone nodules were dissolved in 0.1N sodium hydroxide and quantified using an Epoch microspectrophotometer (BioTek) by an absorbance at 548 nm. All experiments were performed with *n* = 3 human samples per anatomic depot and in triplicate wells (biologic and technical triplicate).

### Adipogenic differentiation assays.

CD146^+^ adipose pericytes from 3 to 8 passages were seeded in 12-well plates at a density of 2 × 10^5^ cells per well and allowed to adhere overnight. At 24 hours after seeding, the basal medium was replaced with adipogenic differentiation medium and replenished every 3 days (Mesencult Adipogenic Differentiation medium, StemCell Technologies Inc.). Oil Red O staining was performed after 10 days of differentiation (4, 40, 42). Cells were washed with PBS and fixed with 4% formaldehyde for 15 minutes. After fixation, cells were washed with water and 500 μL of Oil Red O staining solution. Oil Red O stock solution was prepared by dissolving 0.5 g of Oil Red O in 100 mL isopropanol. Oil Red O staining solution was prepared by dilution of a stock solution with distilled water in a 3:2 ratio, followed by filtration. Oil Red O staining was performed for 30 minutes at 37°C. Following incubation, cells were washed with tap water, followed by microscopy. After imaging, Oil Red O stain was extracted with 100% isopropanol for 5 minutes followed by an absorbance at 548 nm for quantification. All experiments were performed with *n* = 3 human samples per anatomic depot and in triplicate wells (biologic and technical triplicate).

### Proliferation assays.

Proliferation assays were performed in 96-well plates (2 × 10^3^ cells/well) and measured for up to 72 hours using the CellTiter96 AQueous One Solution Cell Proliferation Assay kit (MTS, G358A; Promega) (44). Briefly, 20 μL of MTS solution was added to each well and incubated for 1 hour at 37°C. The absorbance was assayed at 490 nm using an Epoch microspectrophotometer.

### qRT-PCR.

Specific gene expression among CD146^+^ adipose pericytes was assayed by qRT-PCR at 0, 3, and 7 days of osteogenic/adipogenic differentiation, as adapted from prior methods (38, 42, 44). The frequency of CD146^+^CD31^–^CD45^–^ pericytes by anatomic depot of origin and percentage of CD146^+^CD31^–^CD45^–^ of total FACS events were reported in the previous study (4). Total RNA was extracted using TRIzol. In total, 1 μg of total RNA from each sample was subjected to first-strand cDNA synthesis using the iScript cDNA Synthesis Kit (Bio-Rad) to a final volume of 20 μL. The reverse transcription reaction was performed at 25°C for 5 minutes, followed by 46°C for 20 minutes and 95°C for 1 minute. For qRT-PCR, the reaction was performed using 2× SYBR green RT-PCR master mix and a QuantStudio 5 Real-Time PCR system instrument (Thermo Fisher Scientific). qRT-PCR was performed using 384-well optical plates at 95°C for 10 minutes, followed by 40 cycles at 95°C for 15 seconds and at 60°C for 60 seconds. The relative quantification of gene expression was performed using a threshold cycle method according to the manufacturer’s protocol and was normalized to the expression levels of the housekeeping gene *GAPDH* in each sample. Primer sequences are shown in Supplemental Table 2.

### Cell morphology.

Pericytes were seeded at 1 × 10^5^/mL to visualize single-cell populations. After culturing cells for 48 hours, cells were fixed with 4% formaldehyde and incubated with a TIAM1 primary antibody overnight. This was followed by staining with the secondary antibody and Oregon green 488 phalloidin (Thermo Fisher Scientific) for 30 minutes at RT. Afterward, mounting medium with DAPI was applied before covering the Chamber slide (Thermo Fisher Scientific) with glass coverslips. Images of the stained pericytes were captured and analyzed using a ZEISS LSM 800 Confocal Microscope. The intensity of TIAM1 immunostaining was evaluated as mean fluorescence intensity across 5 different random microscopic areas at 63× original magnification. Cellular morphology, including width and length of individual cells from 5 different random microscopic areas at 63× original magnification, was calculated using ImageJ software (1.47v, NIH).

### Intramuscular implantation.

Animals were housed and experiments were performed in accordance with institutional guidelines at JHU. All animal experiments were performed according to JHU Animal Care and Use Committee–approved protocols (MO19M266). DBX (courtesy of Musculoskeletal Transplant Foundation, Edison, New Jersey, USA) was used for the ectopic bone formation assay in mice. Briefly, human CD146^+^ adipose pericytes were pretreated with siRNA for KD or plasmid for overexpression 48 hours before implantation. The scramble siRNA and control plasmid served as controls compared with the TIAM1 siRNA KD and TIAM1 plasmid overexpression. Cells were resuspended at a density of 7.5 ×10^7^ cells/mL in PBS. For each implantation sample, 40 μL of cell suspension was mixed mechanically with 50 mg of DBX allograft putty (3 million total cells in 40 μL PBS) and implanted intramuscularly into the thigh muscle pouch of 10-week-old male athymic mice weight 28~33 g (The Jackson Laboratory). A single implant was placed per mouse. *N* = 4 mice per treatment group, for a total of 16 mice, were used in the experiment. Briefly, animals were anesthetized by isoflurane inhalation and premedicated with buprenorphine. Incisions in the bilateral hind limbs were made, and pockets were cut in the biceps femoris muscles parallel to the muscle fiber long axis by blunt dissection (45). Dissection methods and the surgical manipulation of tissues were kept as constant as possible across animals. After implantation of the cell + scaffold composite, the fascia overlying the muscle was sutured with a simple continuous pattern, and the skin was closed in a separate layer using 5-0 Vicryl (Ethicon).

### μCT imaging and analysis.

Tissues were harvested 8 weeks after implantation and fixed in 4% formaldehyde for 24 hours, transferred to PBS, and scanned using high-resolution μCT (SkyScan 1275; Bruker MicroCT N.V) at an image resolution of 15 μm with the following settings: aluminum filter of 1 mm, x-ray voltage of 65 kV, anode current of 153 μA, exposure time of 160–218 ms, frame average of 6, and rotation step of 0.3° (4). Three-dimensional images were then reconstructed from the 2-dimensional x-ray projections by implementing the Feldkamp algorithm using a commercial NRecon software package (2.0.4.0 SkyScan). For the 3-dimensional morphometric analyses of images, CTVox and CTAn software (1.13 SkyScan) were used. Volumes of interest were shaped by polygons to cover the new bone around the femur with a threshold of 65. The amount of bone formation was analyzed and quantified.

### Statistics.

Statistical analyses were performed in GraphPad Software 6.0. Quantitative data are expressed as mean ± 1 SD. All data were normally distributed. A 2-sample Student’s *t* test was used for 2-group comparisons, and 1-way ANOVA test was used for comparisons of 3 or more groups, followed by Tukey’s post hoc test.

### Study approval.

All animal procedures were approved by IACUC of the JHU. Human samples were obtained under approval from the JHU IRB with a waiver of informed consent.

## Author contributions

GCYH, AZL, and MAGS were responsible for collection and assembly of data, data analysis and interpretation, and manuscript writing. YW, AZL, DL, SN, KB, C Meyers, JX, SW, BP, and C Morris were responsible for collection and assembly of data. AWJ was responsible for provision of study material, conception, design, financial support, manuscript writing, and final approval of the manuscript.

## Supplementary Material

Supplemental data

## Version 1 05/23/2023

In-Press Preview

## Version 2 07/10/2023

Electronic publication
