# Supplementary material for: TIAM1 acts as an actin organization regulator to control adipose tissue–derived pericyte cell fate
Source: JCI Insight. 2023 Jul 10;8(13):e159141. doi: 10.1172/jci.insight.159141 (PMC10371340; doi:10.1172/jci.insight.159141)
Supplement: Supplemental data [file jciinsight-8-159141-s016.pdf]

Supplemental Fig 1.

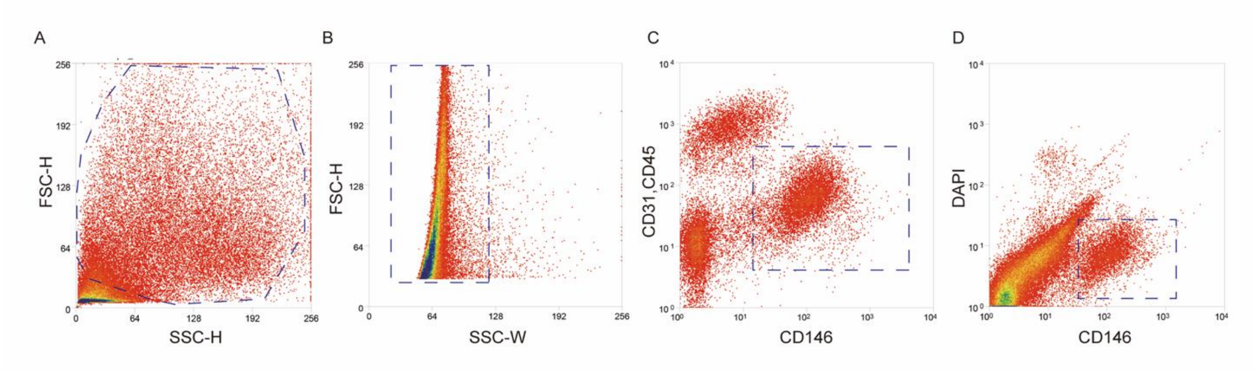

Supplemental Figure 1. Representative FlowJo plots to demonstrate partitioning $CD146^{+}CD31^{-}CD45^{-}$ pericytes from human adipose tissue by Fluorescence activated cell sorting (FACS). (A) Cells from SVF (blue box) are selected on an initial FSC v SSC dot plot. **(B)** Following selection of cell morphology (blue box), **(C)** removal of haematopoietic ($CD45^{+}$) and endothelial cells ($CD31^{+}$) and **(D)** dead cells, pericytes (blue box) were selected based on their immunophenotype ($CD146^{+}CD31^{-}CD45^{-}$).

Supplemental Figure 2.

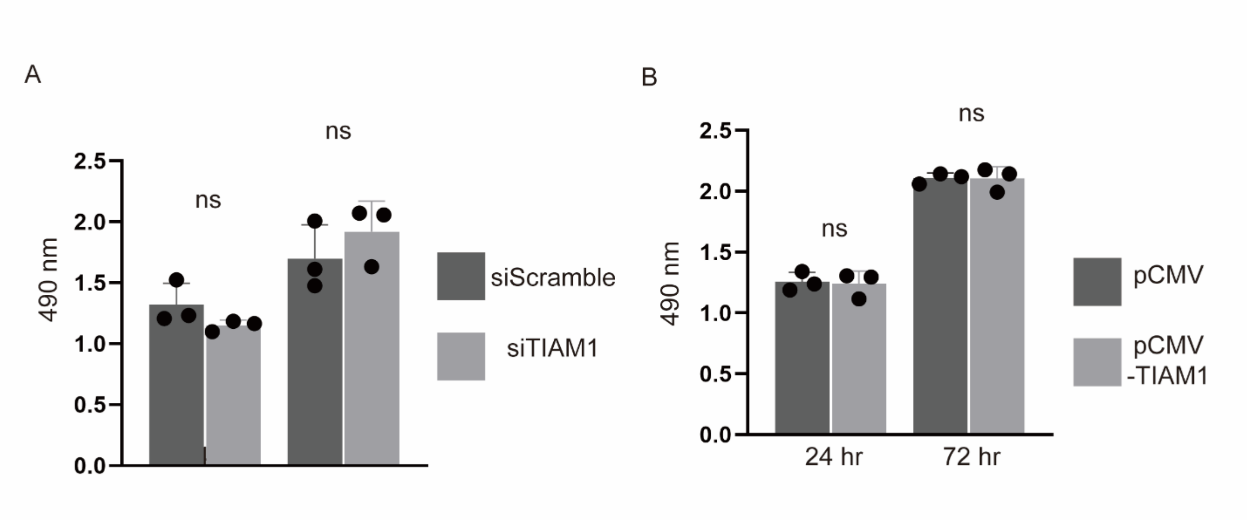

Supplemental Figure 2. No change in proliferation with *TIAM1* misexpression in human pericytes. (A,B) Human adipose tissue pericytes were examined for changes in proliferation after TIAM1 misexpression. MTS assay at 24 and 72 hrs after **(A)** TIAM1 knockdown in comparison to scramble siRNA, and **(B)** TIAM1 overexpression in comparison to pCMV vector control. Ns: non-significant.

Supplemental Figure 3.

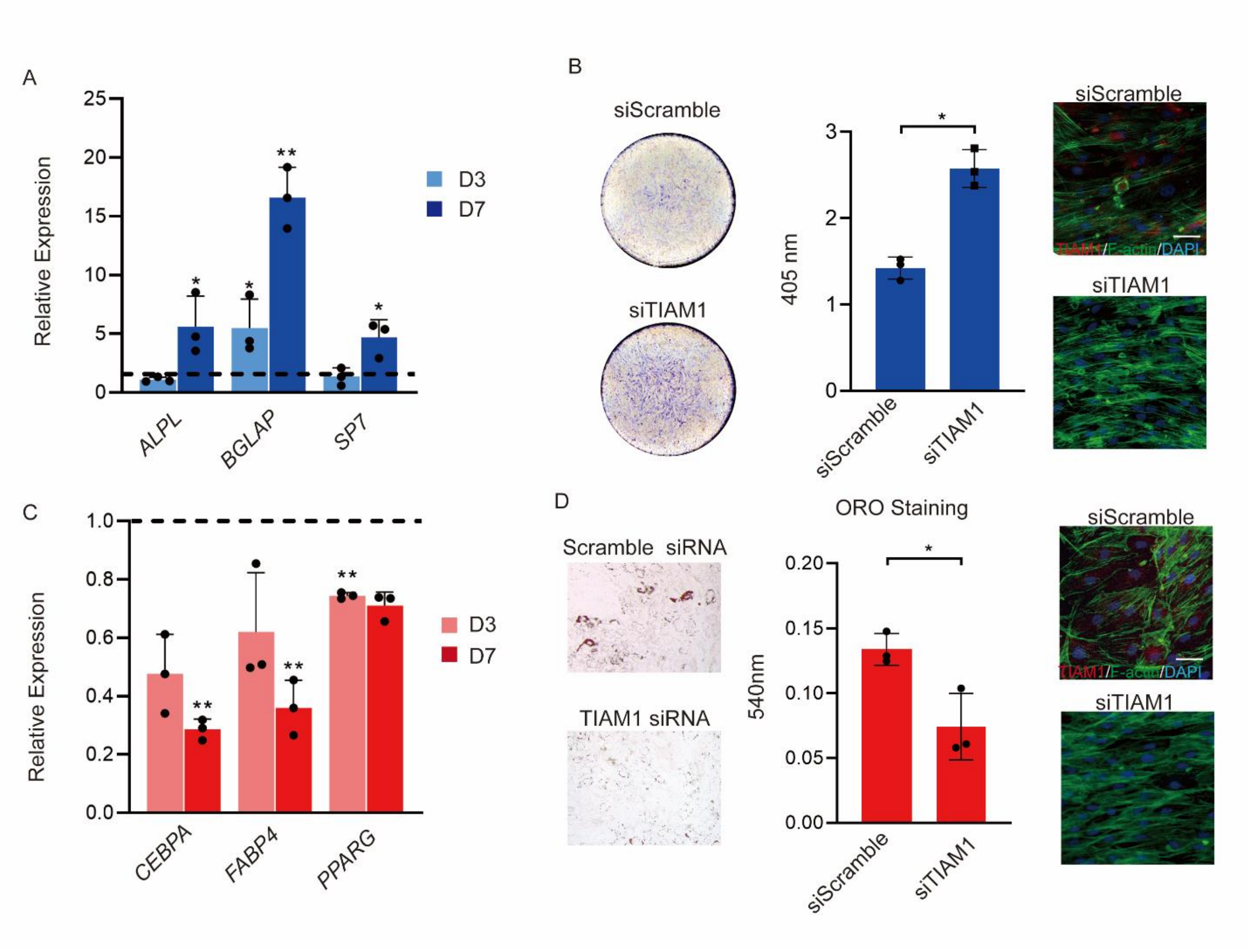

Supplemental Figure 3. *TIAM1* knockdown favors osteogenic over adipogenic differentiation among human periosteal-derived CD146$^+$ pericytes. **(A)** Osteogenic gene markers by qRT-PCR of periosteal pericytes increased on d 3 and 7 after *TIAM1* KD in osteogenic differentiation medium. Dashed lines indicate expression among scramble siRNA at the same timepoint. *ALPL: Alkaline Phosphatase; BGLAP: Osteocalcin; SP7: Osterix*. **(B)** ALP staining, d 10 of differentiation. **(C)** Adipogenic gene marker expression by qRT-PCR with or without *TIAM1* KD during adipogenic differentiation medium, d 3 and 7. Dashed lines indicate expression among scramble siRNA at the same timepoint. *CEBPA: CCAAT Enhancer Binding Protein Alpha; FABP4:*

fatty acid-binding protein 4; PPARG: peroxisome proliferator-activated receptor. **(H)** Oil red O staining and quantification, d 10 of differentiation. *$P<0.05$; **$P<0.01$. Statistical analysis was performed using a one-way ANOVA followed by Tukey’s post hoc test. Representative images shown at 10x magnification.

Supplemental Table 1: Antibodies used.

Antibody	Company	Catalog #	Use
Mouse anti-Human CD31	BD Pharmingen	563653	FACS
Mouse anti-Human CD45	BD Pharmingen	557833	FACS
Mouse anti-Human CD146	Bio-Rad	MCA2141F	FACS
Mouse anti-CD31	Cell Signaling Technology	3528S	IF
Armenian hamster anti-CD31	Abcam	ab119341	IF
Rabbit anti-CD146	Abcam	ab75769	IF / IHC
Mouse anti-CD146	Abcam	ab24577	IF
Rabbit anti- α-SMA	Abcam	ab5694	IF
Rabbit anti-TIAM1	Abcam	Ab211518	ICC/IHC
Sheep anti-TIAM1	R&D system	af5038	ICC
Mouse anti-Human Nuclei	Sigma-Aldrich	MAB1281	IF
Rabbit anti-human Osteocalcin (OCN)	Abcam	ab93876	IF
Rabbit anti-mouse Perilipin 1 (Plin1)	Abcam	ab3526	IF
Donkey anti-Sheep IgG(H+L)	Abcam	ab150179	IF
Goat anti-Mouse IgG(H+L)	Abcam	ab150113	IF
Goat anti-Armenian hamster IgG (H+L)	Abcam	ab173004	IF
Goat anti-rabbit IgG(H+L)	Vector Laboratories	DI1594	ICC / IF
Goat anti-mouse IgG(H+L)	Abcam	ab150119	ICC / IF
FACS: Fluorescent activated cell sorting; ICC: Immunocytochemistry; IF: Immunofluorescent staining; IHC: Immunohistochemistry			

Supplemental Table 2. **Human qRT-PCR primer sequences.**

Gene	Forward 5'-3'	Reverse 5'-3'
GAPDH	CTGGGCTACACTGAGCACC	AAGTGGTCGTTGAGGGCAATG
ALPL	ACCACCACGAGAGTGAACCA	CGTTGTCTGAGTACCAGTCCC
BGLAP	CACTCCTCGCCCTATTGGC	CCCTCCTGCTTGGACACAAAG
SP7	CCTCTGCGGGACTCAACAAC	AGCCCATTAGTGCTTGTAAAGG
CEBPA	TGGACAAGAACAGCAACGAGTA	ATTGTCACTGGTCAGCTCCAG
PPARG	GACAGGAAAGACAACAGACAAATC	GGGGTGATGTGTTTGAACTTG
FABP4	ACGAGAGGATGATAAACTGGTGG	GCGAACTTCAGTCCAGGTCAAC
TIAM1	CCATGAGCAGGGCAGTGT	CGGAGACGGCATCAGAAT
ARPC2	GAACCTCCTCTGGAGCTGAAAG	GAACGTGTGGATCAGGTTGATGG
SRF	TCACCTACCAGGTGTCGGAGTC	GTGCTGTTTGGATGGTGGAGGT

Supplemental Table 3: List of genes shown in Figure 1B.

ACTB	CDH5	FRMD4A	NPHP3	SHTN1
ACTR2	CDK5RAP2	FRMD4B	OOEP	SLC9A3R1
ACTR3	CDX2	FSCN2	OPHN1	SNAP91
AMOT	CEP290	FSCN3	PARD3B	SPINT2
AMOTL1	CFL1	GATA3	PARD6A	SPRY1
AMOTL2	CKAP5	GJA1	PARVG	SYNE2
APC	CLASP1	HES5	PRKCZ	SYNE4
AQP1	CORO7	IFT20	RAC1	TCF15
ARF4	CRB1	IGF1R	RHOA	UBXN2B
ARF6	CRB2	ITGB1	RHOBTB3	WNT5A
ARFGEF1	CRB3	KIF2C	RHOC	NDE1
ARHGAP35	CRK	KIF3A	RHOD	SPAG5
ARPC5	CRKL	KIF26B	RHOF	PARVA
ATN1	CRTAM	LAMA1	RHOH	INSC
BCAS3	CTNNA1	LHX2	RICTOR	
BCCIP	CYP26B1	LIN7A	RIPOR2	
BRSK1	DOCK7	LIN7B	RND1	
BRSK2	DOCK8	LIN7C	RND2	
CAMSAP3	EYA1	MACF1	RND3	
CARMIL2	EZR	MAP1B	RNF41	
PATJ	FAM89B	MAP2	RUFY3	
CCL19	FBF1	MISP	SAPCD2	
CCR7	FEZ1	MSN	SDCCAG8	
CD3G	FGF10	NCKAP1	SH3BP1	
CDC42	FLOT2	NDEL1	SHH	

Supplemental Table 4: List of genes shown in Figure 1C.

ABL1	CDC42EP2	ILK	NF2
ACTA1	CDC42EP5	ITGA4	NRP1
ACTN4	CIB1	ITGAV	NTN4
ANG	CRKL	KIF14	NTNG1
AP1AR	DMTN	LAMB2	NTNG2
APOA1	DNM2	LAMC1	OLFM4
ARAP1	DOCK1	LATS1	P4HB
ARF6	DOCK5	LIMA1	POSTN
ARHGDIB	EPHA1	LIMK1	PREX1
ARHGEF10L	FGA	LLGL1	PXN
ARHGEF17	FGB	LPXN	RAB1A
ARHGEF7	FGD1	MDK	RAC1
ARPC1A	FGD2	MEGF9	RAC3
ARPC2	FGD3	MRAS	RACGAP1
ARPC4	FGD4	MTSS1	RADIL
ARPC5	FGD5	MYADM	RASA1
ATRN	FGD6	MYH11	RHOA
C1QBP	FGG	MYH9	RHOF
CALR	FLNA	MYOC	RHOJ
CAPN1	FLNB	MYOZ1	RICTOR
CAPN2	FN1	NCK1	RND1
CAPNS1	FSCN1	NCK2	RND3
CARMIL1	FSCN2	NEBL	ROCK1
CASS4	GSN	NEDD9	RREB1
CDC42	HAS2	NF1	S100A10
